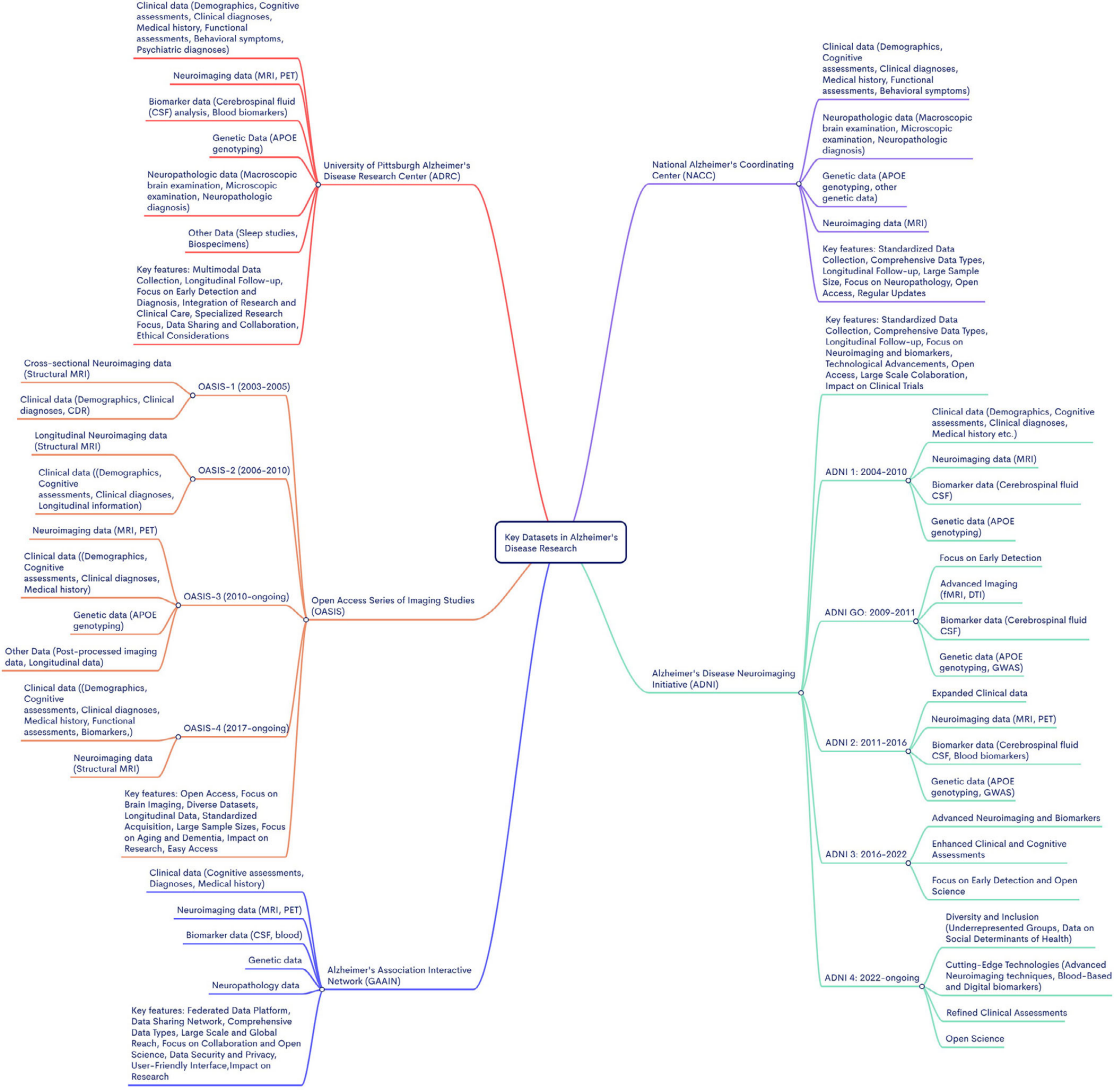# Addressing Heterogeneity, Bias, and Analytical Challenges of Datasets in Alzheimer's Disease Research – A Comprehensive Review

**DOI:** 10.1002/alz70856_103324

**Published:** 2025-12-26

**Authors:** Vinu Sherimon, Abraham Varghese, Sherimon P.C.

**Affiliations:** ^1^ University of Technology and Applied Sciences, Muscat, Muscat, Oman; ^2^ University of Technology and Applied Sciences, Alkhuwair, Muscat, Oman; ^3^ Arab Open University, Muscat, Oman

## Abstract

**Background:**

Research on Alzheimer's disease (AD) requires comprehensive data resources to better understand the complex relationships among genetic, environmental, and clinical variables influencing disease onset and progression. This review systematically analyses significant AD datasets, emphasizing their technical attributes, analytical challenges, and methodological factors to enhance research usability in this domain.

**Method:**

We performed a comprehensive review of published literature and data repositories relevant to AD research. Datasets such as ADNI, NACC, OASIS, Clinical Trial Data (A4, LEARN), and open‐access repositories (AD, Knowledge Portal) were examined. The evaluated key characteristics comprised sample size, data modalities (neuroimaging, genomics, proteomics, clinical, longitudinal coverage, data access policies, and identified constraints).

**Result:**

Comprehensive initiatives such as ADNI, and NACC contribute essential multimodal data, enabling research on AD biomarkers, progression, and treatment efficacy. Nonetheless, intrinsic issues include:

*Data Heterogeneity*: Inconsistencies in diagnostic criteria, evaluation methodologies, and imaging modalities among studies impede data synchronization and comparability (e.g., MCI diagnosis inconsistencies between NACC and ADNI)

*Missing Data*: Incomplete datasets require precise management of missing values to prevent skewed analysis. Sophisticated techniques for imputation and sensitivity analysis are essential.

*Class Imbalance*: Unequal representation of diagnostic categories (e.g., normal, MCI, AD) might affect the efficacy of machine learning models, necessitating approaches such as data augmentation (SMOTE) or cost‐sensitive learning.

*High Dimensionality*: The integration of multiomics data requires feature selection techniques (such as genetic algorithms and modified particle swam optimization) to determine the most significant aspects and mitigate computational complexity.

**Conclusion:**

Despite the above limitations, current AD datasets have contributed to significant advancements. Future research should focus on:

*Standardization*: Supporting uniform data gathering and processing techniques across research initiatives.

*Data Integration*: Formulating effective strategies for integrating multi‐omics, neuroimaging, and clinical data to explain the complex relationships of variables driving AD.

*Advanced Analytics*: Implementing complex machine learning methodologies to address class imbalance, missing data, and high dimensionality while ensuring model interoperability and generalizability.

*Open Science*: Promoting open data sharing to enhance collaborative research and optimize data value.

This review underlines the necessity for continuous initiatives to enhance data quality, address methodological challenges, and support for open science principles to expedite AD research.